# Tools for primary care patient safety: a narrative review

**DOI:** 10.1186/1471-2296-15-166

**Published:** 2014-10-26

**Authors:** Rachel Spencer, Stephen M Campbell

**Affiliations:** Division of Primary Care, School of Community Health Sciences, University of Nottingham Medical School, Queen’s Medical Centre, Nottingham, NG7 2UH UK; NIHR Greater Manchester Primary Care Patient Safety Translational Research Centre, University of Manchester, 7th Floor, Williamson Building, Manchester, M13 9PL UK; Centre for Primary, Institute of Population Health, University of Manchester, 7th Floor, Williamson Building, Manchester, M13 9PL UK

**Keywords:** Family practice, Patient safety, Review

## Abstract

**Background:**

Patient safety in primary care is a developing field with an embryonic but evolving evidence base. This narrative review aims to identify tools that can be used by family practitioners as part of a patient safety toolkit to improve the safety of the care and services provided by their practices.

**Methods:**

Searches were performed in 6 healthcare databases in 2011 using 3 search stems; location (primary care), patient safety synonyms and outcome measure synonyms. Two reviewers analysed the results using a numerical and thematic analyses. Extensive grey literature exploration was also conducted.

**Results:**

Overall, 114 Tools were identified with 26 accrued from grey literature. Most published literature originated from the USA (41%) and the UK (23%) within the last 10 years. Most of the literature addresses the themes of medication error (55%) followed by safety climate (8%) and adverse event reporting (8%). Minor themes included; informatics (4.5%) patient role (3%) and general measures to correct error (5%). The primary/secondary care interface is well described (5%) but few specific tools for primary care exist. Diagnostic error and results handling appear infrequently (<1% of total literature) despite their relative importance. The remainder of literature (11%) related to referrals, Out-Of-Hours (OOH) care, telephone care, organisational issues, mortality and clerical error.

**Conclusions:**

This review identified tools and indicators that are available for use in family practice to measure patient safety, which is crucial to improve safety and design a patient safety toolkit. However, many of the tools have yet to be used in quality improvement strategies and cycles such as plan–do–study–act (PDSA) so there is a dearth of evidence of their utility in improving as opposed to measuring and highlighting safety issues. The lack of focus on diagnostics, systems safety and results handling provide direction and priorities for future research.

**Electronic supplementary material:**

The online version of this article (doi:10.1186/1471-2296-15-166) contains supplementary material, which is available to authorized users.

## Background

Patient safety has been on the agenda of hospital physicians since the publication of the Institute of Medicine’s 2000 report, ‘To Err is Human’, revealed that more people were dying in the US as a result of medical error than from road traffic accidents [1]. However, most healthcare interactions in the developed world occur in family medicine: 90% of contacts in the England with the National Health Service take place in primary care [2]. In England there are approximately 1 billion community prescriptions dispensed each year [3]. The potential for adverse events is therefore huge but the knowledge base about primary care patient safety is still sparse. A literature review of the nature and frequency of error in primary care suggested that there are between 5–80 safety incidents per 100,000 consultations, which in the UK would translate to between 37–600 incidents per day [4]. Another review estimates that there may be a patient safety incident in approximately 2% of family practice consultations [5].

A 2011 report by the American Medical Association on ambulatory patient safety concluded that the introduction of, and research into, patient safety in the primary care environment have lagged behind that of secondary care [6]. Understanding the epidemiology of hospital errors was crucial in developing hospital based safety interventions and the media’s reporting of this data ensured public support for efforts to improve safety [6]. Some of its authors concluded that there needed to be a similar focus on primary care, because there were ‘virtually no credible studies on how to improve safety’ [7]. Moreover, a report by the Health Foundation in 2013 emphasised the importance of knowing what methods, tools and indicators are currently being used in primary care to measure patient safety [8]. In this paper, patient safety refers to the ‘avoidance, prevention and amelioration of adverse outcomes or injuries stemming from the process of healthcare’ [8].

Staff and systems in primary care environments have the potential to contribute to serious error that can cause both morbidity and mortality; which has been demonstrated in the field of prescribing [9]. Evidence on primary care error comes mainly from the statistics of the medical defence organisations and from small pilot studies [10]. And yet, experts we have consulted in the field were anecdotally aware of a multiplicity of interventions or ‘tools’ from their own and others’ work world-wide, which helped identify grey literature for this study.

This paper reports a narrative review of ‘tools’ to improve, measure, and monitor patient safety in the ambulatory settings with a focus on family practice. A narrative review broadly covers a specific topic but does not adhere to strict systematic methods to locate and synthesize articles and enables description and synthesis of qualitative research and categorises studies into more homogenous groups [11]. To the authors’ knowledge no such broad-ranging review has been attempted. The context of this study is worldwide including both the US and the UK and throughout the term primary care is used to address the terms general practice and family practice.

## Methods

### Data sources and searches

Our structured narrative review was planned and conducted according to guidance in the Preferred Reporting Items for Systematic Reviews and Meta- Analyses (PRISMA) guidelines [12] but following a more narrative approach (especially with regard to grey literature). The starting point for determining the search terms used in the review was a 3 point definition of our search question and exploration of Medical Subject Heading (MeSH) terms [13]. We used a multi-centre team (including leading UK experts on patient safety) at a strategic planning meeting to comment on and finalise the search terms for the review. References were managed in *Endnote.* Broad ranging search terms were used for developing a search strategy in 3 stems; setting (primary care [i.e. general/family practice, ambulatory care, community care, generalist care], safety synonyms [i.e. error, adverse event, fault, malpractice] and types of tool [i.e. indicator, survey, guideline, scale] (see web Additional file 1: Appendix 1). The aim was to be as inclusive as possible and address administrative, clinical and patient experience issues. The search was performed on the following databases; Embase, CINAHL, Pubmed, Medline (Ovid 1996 onward), Health Management Information Consortium and Web of Science on the 1/11/2011. We did not limit our search by year of publication or to the English language, in order to capture a world-wide perspective on patient safety. However, only abstracts in English were included due to resource restraints for translation. Grey literature was identified from known internet patient safety sources from the US and UK to expand the scope of the review (see web Additional file: 1 Appendix 6). In order to fully explore a single tool many resources often had to be read – for example the IHI trigger tool is described in a number of web publications and supporting documents. Care was taken not to count published tools that also appeared in grey literature twice by discussion between the two reviewers and wider team.

### Study selection

Reviewer one was a GP Academic Clinical Fellow with an interest in pharmacology (RS) and reviewer two was a health services researcher with an interest in family practice (SC). Similarly to the strategy used in the AMA’s report [6], we were interested in highly generalisable tools so research addressing single drugs or conditions in very specific settings was excluded. Inclusion and exclusion criteria are listed in below. An inclusive strategy was employed such that neither reviewer could exclude studies the other felt were potentially relevant. Disagreements were resolved by regular discussions between the 2 reviewers. The inclusion and exclusion criteria were as follows:

- Exclusion criteria:
  - hospital care/setting - unless transferable
  - opinion pieces/editorials
  - single drugs/conditions where the focus was felt to be on the specific drug or condition rather than on transferable tools
  - only about quality of care without explicit patient safety component
  - exclude on basis of journal (for example “Health Care Food & Nutrition focus”)
  - economic impact of errors (relevant papers were taken out at this stage for other purposes within the project)
  - Both the abstract and main text were not in English
- Inclusion criteria:
  - if unsure always include - for example, ‘good advice’ which might later inform other tools
  - tools or strategies to improve or analyse safety which are of relevance to Primary Care.

### Data quality and extraction

Data were extracted independently by the two reviewers (RS and SC). A dual approach was taken to data extraction from published material using both a *Word* document (Web Additional file: 1 Appendix 5) and an *Excel* template. For reporting data from selected papers we used a modified PRISMA [12] checklist, which combined aspects of different methodologies (not just systematic reviews and meta-analyses) into a form for all study types (available on application to the authors). For example the PRISMA checklist requires a discussion of limitations which is a highly transferable requirement to all methodologies, but it also requires specific methods based items such as ‘confidence intervals on meta-analyses’. Using information collected on the modified PRISMA form, a numerical data extraction system was agreed by both reviewers in order to present results from the selected papers data for analysis were extracted from that *Excel* document. A pilot of 10 key papers with differing methodologies was undertaken prior to commencing full data extraction – high levels of agreement were found between the two reviewers. At the end of data extraction differences in rating on the *Excel* sheet were discussed and analysed across a series of face-to-face and telephone meetings, attempts to reach consensus were almost always possible.

### Funding

This review is part of a National Institute for Health Research (NIHR), School for Primary Care Research (SPCR: http://www.spcr.nihr.ac.uk/) (UK) project, undertaken with the aim of constructing a Patient Safety Toolkit for English family practice.

## Results and discussion

**Figure 1:**
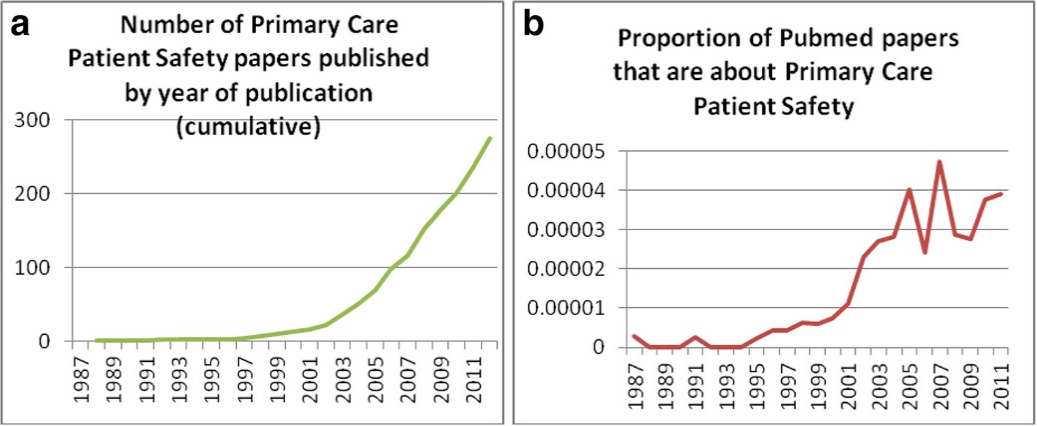
**‘Toolkit’ review stages.** Graph 1 –**a)** illustration of the literature base in primary care patient safety 1987-2011from Pubmed **b)** Papers from the review divided by the annual Pubmed output for the same year.

Grey literature results are not included in the following calculations and flow diagrams; results are instead included in the list of tools found in web Additional file 1: Appendix 3 (where they are clearly marked as being from grey sources). Using the process described in Figure 1, we selected approximately 10% (n = 1311) of the original search total (n = 13,240) for evaluation of abstracts; titles excluded at this stage were clearly not of relevance e.g. relating to non-healthcare safety topics. Abstracts were then analysed for tools, after excluding papers which were from the correct setting but which did not contain any interventions; around 14% of the abstracts were included for full paper analysis (n = 189).

As Graph 1a) illustrates, the majority of publications in the review have been published in the last decade. These data could be a product of MeSH term development or consistency in the last 10 years. However, as Graph 1b) shows, the same take-off in 2001 occurs even when presented as a proportion of the total literature published on *Pubmed*. Analysing the MeSH terms of the 11 papers in the review from prior to 2000 revealed that the terms ‘medical error’ and ‘diagnostic error’ were each used twice and ‘risk management’ was used in 3 papers. Keywords using the term ‘drug’ appeared 13 times, family practice and ambulatory care were commonly used keywords.

The majority of the literature focused on prescribing (55%), which excludes IT interventions for prescribing that were attributed to the informatics theme instead. Other prominent areas were adverse events in primary care and safety climate (which comprised 8% of the total published literature each). A number of climate measures had been refined from earlier climate surveys for use specifically in primary care (i.e. SAQ (ambulatory) [14]. Minor themes included; informatics (4.5%) patient role (3%) and general measures to correct error (5%). The primary/secondary care interface is well described in the literature (5%) but, as yet, only 1 published interface tool specifically for primary care exists. Diagnostic error and results handling appear infrequently (<1% of total literature) despite their relative importance. The remainder of literature (11%) related to referrals, Out-Of-Hours (OOH) care, telephone care, organisational issues, mortality and clerical error. Overall, 114 Tools were identified with 26 accrued from grey literature.

The setting of the research uncovered was predominantly family practice (in keeping with our search strategy); the term ‘health system’ was used to describe research such as consensus outputs from multi-disciplinary teams or across the whole healthcare system. Most published literature was US based (41%) followed by UK located studies (23%), with other countries producing no more than 5% of published papers each. The grey literature also reflects the predominance of US and UK sources. A variety of study designs were identified in the review including, for example, consensus techniques (10%) , observational methods (15%) but most were mixed methods studies in patient safety research.

We classified the data from the published papers in this review using a taxonomy for primary care patient safety based on previous taxonomies by experts in quality of care and patient safety [15–19]. It differs from our data collection form as it was evolved later in the process of our analyses. In our taxonomy (Web Additional file 1: Appendix 4) there are two principal dimensions of safety: ‘access to safe services’ and ‘effectiveness of safety processes’, which are discussed in terms of the structure of the health care system, processes of safety and health outcomes. This taxonomy is based on previous conceptual work on quality of care [15]; in essence, do users get the safe care they need, and is the care safe when they get it?

In our review, 88 of the 114 unique tools identified (Web Additional file 1: Appendix 3) came from the published literature and 26 from grey literature sources – the majority of these being from the websites of known patient safety organisations (see data sources in Methods section). The review identified a wide range of ‘tools’ that cannot be described fully here due to word count constraints. The detailed output from the review will be described across a series of subsequent papers on ambulatory patient safety. However, Table 1 shows key examples found in each dimension of patient safety: We have presented the most well-known or most often used US or UK tools as illustrative examples. The Table is presented in order of weight of literature with most common topics appearing first.

**Table 1:** **Types of tools found in the review, where possible well-known US examples of the type of tool are given in order to aid understanding**

Type of tool (Explanation of tool)	Used in the US?	Used in the UK?	US Example	Data source	Number of primary care tools of this type identified
Prescribing Indicator Packs	Yes	Yes	Beers criteria [20]	EHR	15 main sets, much overlap -3
(criteria for ‘never events’ in prescribing) - other prescribing tools			GRAM reports [21], MAI [22] (Geriatric Risk Assessment MedGuide™ Medications Appropriateness Index)	EHR, staff	
Trigger Tools					
-General	Yes	Yes	IHI Outpatient Adverse	EHR	5
-Medications	Yes	No	Event Trigger Tool [23]		3
-Surgery	Yes	Yes	Adverse drug events among older adults in primary care [24] Ambulatory surgery [25]		1
*(Criteria are screened for in a sample of medical records ‘triggering’ more detailed review)*					
Event Reporting Systems (National systems for informing relevant authorities about safety problems with all aspects of healthcare)	Yes	Yes	ASIPS [26] (Applied Strategies for Improving Patient Safety)	EHR, Staff and patients	6
Medicines/device Reporting Systems	Yes	Yes	MEADERS [27]	EHR, Staff and Patients	4
(National systems for informing relevant authorities about safety problems specific to the above)			(Medication Error and Adverse Drug Event Reporting System) VAERS [28] (Vaccine Adverse Events Reporting System)		
Safety Climate/Culture Measures (The practice team rate themselves against safety criteria and discuss the results to make changes)	Yes	Yes	Safety Attitudes Questionnaire [14]	Staff	10
Significant Event Analysis Tools (The practice team discuss untoward events, using a standardised structure, in order to learn from them)	No	Yes	UK example - NHS Education for Scotland [29]	Staff, EHR and patients	5
General Primary/Secondary Interface Tools (standardised systems for handling patient care at transition in care level – often electronic discharge summaries)	Yes	Yes	‘Care Transitions Approach’ [30]	EHR, hospital records	Only 3 within the direct control of family doctors
Medication Reconciliation Tools (aligning medication histories after secondary care contact)	Yes	No formal tool used	Partner’s Post Discharge Tool [31]	EHR, hospital records	3
PROMs for safety (questionnaire determining the patient perspective of safety in their practice)	Yes	Yes	SEAPS [32] (Seniors Empowerment and Advocacy in Patient Safety)	Patients	8
Other Patient Involvement Measures (variety of tools including literature for patients, computerised systems and medications specific tools)	Yes	Yes	‘Speak-Up’ from JCAHO [33]	Patients	4
IT Measures	Yes	Yes	SEMI-P [34]	EHR	11
(not just CDSS but a variety of measures often tackling systems error, many relate to prescribing safety)			(Safety Enhancement and Monitoring Instrument that is Patient centred)		
Diagnostic Tools (Mainly CDSS designed to improve diagnosis)	Yes	No	DxPlain [35]	EHR	3

*Abbreviations*:

*CDSS* Computer Decision Support Software.

*EHR* Electronic Health Record.

*PROM* Patient reported outcome measures.

*UK* United Kingsdom.

*US* United States.

### Summary of main findings

We have demonstrated that there has been an upsurge in publications on primary care patient safety since 2001 and that most of the literature comes from the USA and the UK, with the pre-eminent topic being prescribing safety. The list of discrete tools (which includes grey literature) has a much more even spread across the dimensions within our conceptual taxonomy (Web Appendices 3 and 4). Using this taxonomy shows that some areas of patient safety are relatively neglected in the published literature on primary care patient safety tools; for example, diagnostic error. Tools for test results and referrals are also poorly represented; there were 5 descriptive papers in total, one un-validated tool for electronic referrals and one indicator set dealing with referrals from OOH care. No tools for investigations management were found.

### Comparison to existing literature

To the authors’ knowledge no similar review has been undertaken to look specifically at instruments for measuring patient safety in primary care. The AMA report on ambulatory patient safety [6] found that the number of reported interventions in primary care is low but their search strategy did not take a worldwide approach and only focused on interventions that reduce error or harm. We designed a more inclusive search strategy to capture measurement tools and strategies and were therefore able to find a wider body of literature. The focus on measurement tools and strategies reflects the importance of knowing what is being used currently in primary care to measure patient safety [8]. Many of the 114 tools found are iterations of tools constructed previously and re-designed for other countries. For example, the UK NHS Institute for Innovation and Improvement Primary Care Trigger Tool [36] has much in common with the IHI Outpatient Adverse Event Trigger Tool [23].

Our study resonates with the view that there can be no one single measure of patient safety [8]. Rather, a framework for patient safety should include for example; past harm, reliability, ‘sensitivity to operations’, anticipation/preparedness and Integration/learning. Vincent C, 2013 [8] Measures of past harm are prevalent among the tools we found i.e. – adverse event reporting systems. Some tools clearly straddle boundaries within the definition: Significant Event Analyses (SEA) (a technique commonly used in the UK), for example, straddles past events, future anticipation of similar situations and learning in relation to the significant event. Few tools address safety reliability in primary care; practices may set their own standards for audit of patient safety but as yet no formal targets exist for primary care in the UK or US (in direct contrast with hospital mortality data and target dashboards such as HEDIS - http://www.ncqa.org/HEDISQualityMeasurement.aspx). Sensitivity to operations is an umbrella term referring to the information and capacity in clinical systems to monitor safety on an hourly or daily basis, climate measures often address questions to staff in relation to their adaptability to change but there are no other measures of this dynamic in primary care. The work of the defence organisations in advising practices about risks and loop-holes in operating systems comes close to fulfilling this goal and roughly equates to a safety ‘walk-round’ [37]. The challenge to any ‘toolkit’ is to incorporate prospective measures that prevent and anticipate error. The major elements of the toolkit that address prevention are; trigger tools [23–25, 36] (potential rather than actual harm), medicines reconciliation packages [30] (prevent harm from changes to prescriptions at the interface of primary and secondary care), safety culture [38] and a ‘safe systems’ checklist, which encourages primary care practices to seek out loopholes in their established systems.

### Strengths/limitations

This review presented challenges due to the broad nature of our question and that there are no criteria for a standardised definition of, or criteria for classifying, a ‘tool’. Tools can be alerts, scoring systems, order sets, dashboards, questionnaires, educational materials, forms or templates to name but a few and, as such, the output of the review is highly heterogeneous. Therefore, we employed pragmatic ways of handling the large amount of data extracted from the review. Our strengths have included using a two reviewer system and a dual extraction process of both numerically coded data and free text summaries of papers, which enabled us to analyse identified instruments in-depth and an extensive exploration of grey literature with a world-wide perspective. Exclusions due to translation costs were minor – only 6 papers out of 280 were excluded on language basis alone. Time-constraints on the project meant we could not use back and forward citation methods systematically due to the sheer number of papers involved in the review.

### Implications for practice, policy, or future research

The main aim of the Tools identified is to measure or report safety issues. While measurement and baselines are a prerequisite to improvement, few of the Tools include an embedded implementation strategy that would address improvement or a quality cycle to alter strategies and measure for change [39]. It is difficult to estimate the impact the various measurement tools identified in this review would have in improving patient safety; for instance, prescribing indicators would only seem to measure level of harm at surface value but have been found to change harmful prescribing patterns when combined with educational feedback [40]. Moreover, standards and consistency of reporting vary and many studies, for example around culture and climate surveys, do not report reliability, validity, details of their study characteristics and participants etc.

Others have advocated the need for outcome measures in patient safety [41]. However, measurement systems need to be tested to ensure they measure what is claimed, whether they can reliably tell if deterioration or improvement is occurring and what other (untoward and unintended) consequences could occur [8]. This adheres to the wider imperative that measures of quality or safety, and the data collected, adhere to key attributes such as reliability and validity and also address issues such as acceptability, implementation issues and possible unintended consequences [42, 43]. The aim of future work will be to test the suitability and acceptability of the proposed measures in the toolkit and to test changes within practices after application of the toolkit; as well as intended and unintended (positive and negative) consequences. Measureable outcomes are only one feature of Safety Management Systems, and as such the toolkit should not rely exclusively on them but also develop other areas such as training, policy, culture and feedback of outcomes data in line with other established models of patient safety [9, 43–45]. There is a need also to embrace qualitative methodologies to patient safety such as the Manchester Patient Safety Framework (MaPSaF ) [46].

## Conclusion

We have identified 114 published and unpublished tools and indicators, which can be used currently in primary care to measure patient safety. However, the AMA concluded that there are virtually no credible studies on how to improve safety in primary care [6] andthe challenge is still to turn measurement into improvement as few tools have been used in quality improvement cycles or as part of performance targets for safety in ambulatory care. Having a comprehensive set of tools for tracking and preventing safety events is the first step in fixing that, and this paper clearly shows where our current toolkit is wanting. The results of this review will enable a better understanding of the epidemiology of ambulatory care safety and help underpin the future development of primary care based safety interventions.

## Authors’ information

Dr Rachel Spencer is an academic General Practitioner at the University of Nottingham and Professor Stephen Campbell is a health services researcher at the University of Manchester.

## Electronic supplementary material

Additional file 1: **Tools for Primary Care Patient Safety; a Systematic Review.** (DOCX 65 KB)

## Abbreviations

AMA: American Medical Association
NIHR SPCR: National Institute for Health Research School for Primary Care Research
PRISMA: Preferred Reporting Items for Systematic Reviews and Meta- Analyses
MeSH: Medical Subject Heading
CINAHL: Cumulative Index of Nursing and Allied Health Literature
IHI: Institute for Healthcare Improvement
SAQ (ambulatory): Safety Attitudes Questionnaire
OOH: Out of Hours
SEA: Significant Event Analyses.
