# Supplementary material for: Tools for primary care patient safety: a narrative review
Source: BMC Fam Pract. 2014 Oct 26;15:166. doi: 10.1186/1471-2296-15-166 (PMC4288623; doi:10.1186/1471-2296-15-166)
Supplement: Supplementary file 1 — Additional file 1: Tools for Primary Care Patient Safety; a Systematic Review. (DOCX 65 KB) [file 12875_2014_1143_MOESM1_ESM.docx]

**Appendices for Web Version**

Tools for Primary Care Patient Safety; a Systematic Review

**Web Appendix 1. search terms**

1. Setting

"Family physician" OR "primary care" OR "family practice" OR "general practice" OR "ambulatory care" OR "ambulatory health" OR "ambulatory health-care" OR "ambulatory healthcare" OR "community health" OR "community healthcare" OR "community health-care" OR "primary health" OR "primary healthcare" OR "primary physician" OR "primary health-care" OR generalist OR "family medicine"

1. Safety synonyms

"administration error" OR "administration errors" OR "dispensing error" OR "dispensing errors" OR "medication error" OR "medication errors" OR "medical mistake" OR "medical mistakes" OR "prescription error" OR "prescription errors" OR "prescribing error" OR "prescribing errors" OR "prescribing fault" OR "prescribing faults" OR "medical error" OR "medical errors" OR malpractice OR "safety" OR "safety-culture" OR "adverse event" OR "adverse events" OR "adverse effect" OR "adverse effects" OR "adverse reaction" OR "adverse reactions" OR harm OR harms

1. Types of tools

scale OR scales OR survey OR surveys OR questionnaire OR questionnaires OR instrument OR instruments OR indicator OR indicators OR "outcome assessment" OR "outcome assessments" OR "patient reported outcome" OR "patient reported outcomes" OR "patient experience" OR "patient experiences" OR "practice guideline" OR "practice guidelines" OR "quality assurance" OR tool OR tools OR toolkit OR toolkits

# Appendix 2. Data extraction form (*Word*) with example

| Ref. citation | Summary of aims; method and sample and analyses (extract from abstract where possible) | What is/are the Indicator(s) / results | Main strengths / weaknesses | Adaptable to General Practice Computer Systems? |
| --- | --- | --- | --- | --- |
| Singh et al Identifying diagnostic errors in primary care using an electronic screening algorithm. *Arch Intern Med. 2007;167:302-308* | **Safety focus:** diagnostic  **Aim:** design and test an algorithm for automated detecting of unscheduled return visits to primary care (as a marker for diagnostic error). Also to develop a classification system for diagnostic error  **Methods:** Structured query language (SQL) database used to extract information from computerized medical records in one closed hospital system. For hospitalization 15580 records yielded 211 cases 139 of which met criteria. For repeat visit or Emergency Department attendance 5267 records yielded 175 cases. 199 controls with only 1 visit were chosen at random. Three reviewers were blinded to the aims of the study and used a standardized form to extract data on potential errors. Two physicians reviewed each case independently – diagnostic error was only assigned on the basis of information available to the doctor at the time and not future tests.  **Sample:** A veteran’s affairs clinic with a patient population of 6000. Cases had an index visit followed by either hospitalization or an unscheduled return visit between 24hours and 10 days later. Planned hospital admissions excluded by manual notes review  **Analyses:** Kappas used for agreement between reviewers, p values levels <0.05 used. | **Outcomes:** 24% Positive Predictive Value (PPV) of hospitalization for error, 10% PPV of second visit for error, 4% PPV in controls.  The most common errors in the primary diagnostic process were failure/delay in history taking and misinterpretation of the history and physical examination findings. The most common secondary errors were lack of prioritization/awareness of severity.  This tool has PPVs comparable to other methods for diagnostic error detection and better than those for ADE detection.  Potential criterion; the practice can demonstrate a certain sample of the medical records have been screened and invetigated for diagnostic error | **Limitations:** may not be generalizable outside the Veterans Association setting. These results may apply more to trainee doctors.  **Strengths:** complete blinding by using a standardised tool | Yes - the initial screens were computerized but the later detailed review cannot be computerized |

Web Appendix 3. List of tools arising from the systematic review (grey literature sources are in italics)

| **Taxonomy** | **Safety Focus** | **Title of Tool, Questionnaire, PROM/PREM or Indicator set (with reference)** |
| --- | --- | --- |
| All Areas | General | *AHRQ Medical Office Survey on Patient Safety*  [*http://www.ahrq.gov/qual/patientsafetyculture/mosurvindex.htm*](http://www.ahrq.gov/qual/patientsafetyculture/mosurvindex.htm) |
|  |  | *The CRSA UK (MPS)* [*http://www.medicalprotection.org/uk/education-and-events/clinical-risk-self-assessments-for-GPs*](http://www.medicalprotection.org/uk/education-and-events/clinical-risk-self-assessments-for-GPs) |
|  |  | *Medical Home Accreditation standards (JCAHO)* [*http://www.jointcommission.org/accreditation/primary_care_medical_home_certification_option_for_cah.aspx*](http://www.jointcommission.org/accreditation/primary_care_medical_home_certification_option_for_cah.aspx) |
|  |  | *National Patient Safety Goals (JCAHO)* [*http://www.jointcommission.org/2013_npsgs_slides/*](http://www.jointcommission.org/2013_npsgs_slides/) |
|  |  | Eleven Best practices  Schauberger, C. W. and P. Larson (2006). "Performance improvement. Implementing patient safety practices in small ambulatory care settings." Joint Commission Journal on Quality & Patient Safety **32**(8): 419-425. |
| Structure + Systems | Learning Organiz-ation | The European Practice Assessment (EPA)  Engels, Y., et al. (2005). "Developing a framework of, and quality indicators for, general practice management in Europe." Family Practice **22**(2): 215-222.  *and TOPAS development of EPA* [*www.topaseurope.eu/*](http://www.topaseurope.eu/) |
|  |  | Performance Concerns in Primary Care  Essex, B., et al. (2007). "Performance concerns in primary care : a Delphi consensus on risk and investigation." Quality in Primary Care **15**(5): 293-300. |
|  |  | ‘3 most serious errors’ – a questionnaire for GPs  Fisseni, G., et al. (2008). "Responding to serious medical error in general practice--consequences for the GPs involved: analysis of 75 cases from Germany." Family Practice 25(1): 9-13. |
|  |  | Know Your Own Risk V1  Johnson, M., et al. (2008). "Self-assessment of medico-legal risk by doctors: the Know Your Risk Version I - Short Form." Australian Health Review **32**(2): 339-348. |
|  |  | Practice Accreditation Scheme  Campbell, S. M., et al. (2010). "Primary Medical Care Provider Accreditation (PMCPA): pilot evaluation." Br J Gen Pract **60**(576): 295-304. |
|  |  | Physicians’ Worklife Study Instrument  Linzer, M., et al. (2005). "Organizational Climate, Stress, and Error in Primary Care: The MEMO Study Findings” Advances in Patient Safety: From Research to Implementation (Volume 1: Research Findings). Agency for Healthcare Research and Quality (US); 2005 Feb. |
|  |  | *Incident Decision Tree (National Patient Safety Association)*  [**http://www.nrls.npsa.nhs.uk/resources/?EntryId45=59900**](http://www.nrls.npsa.nhs.uk/resources/?EntryId45=59900) |
|  |  | *Root cause analysis toolkit (National Patient Safety Association)*  [**http://www.nrls.npsa.nhs.uk/resources/rca-conditions/**](http://www.nrls.npsa.nhs.uk/resources/rca-conditions/) |
|  | Climate measures | Safety Attitudes Questionnaire (ambulatory version)^14^ |
|  |  | Primary Care Safe Quest (PC Safe Quest)  de Wet, C., et al. (2010). "The development and psychometric evaluation of a safety climate measure for primary care." Quality & safety in health care **19**(6): 578-584. |
|  |  | *Staff Survey of Patient Safety Culture (MPS – commercial tool)*  *www.medicalprotection.org* |
|  |  | Learning Practice Inventory  Kelly, D. R., et al. (2011). "Diagnosing a learning practice: the validity and reliability of a learning practice inventory." BMJ Qual Saf **20**(3): 209-215. |
|  |  | FraSiK – Frankfurt Patient Safety Climate Questionnaire for GPs  Hoffmann, B., et al. (2011). "The Frankfurt Patient Safety Climate Questionnaire for General Practices (FraSiK): analysis of psychometric properties." BMJ Quality & Safety **20**(9): 797-805. |
|  |  | MaPSaf (Manchester Patient Safety Framework)  Kirk, S., et al. (2007). "Patient safety culture in primary care: developing a theoretical framework for practical use." Quality & safety in health care **16**(4): 313-320. |
|  |  | Safety Climate assessment measure for primary care  Schutz, A. L., et al. (2007). "Development of a patient safety culture measurement tool for ambulatory health care settings: analysis of content validity." Health Care Management Science **10**(2): 139-149. |
|  |  | *TCAM – NPSA*  [***http://www.nrls.npsa.nhs.uk/resources/?entryid45=59884***](http://www.nrls.npsa.nhs.uk/resources/?entryid45=59884) |
|  |  | Teamwork and Safety Climate Survey  Hutchinson, A., et al. (2006). "Use of a safety climate questionnaire in UK health care: factor structure, reliability and usability." Quality & safety in health care **15**(5): 347-353. |
|  |  | SCOPE  Zwart, D. L., et al. (2011). "Patient safety culture measurement in general practice. Clinimetric properties of SCOPE." BMC Fam Pract **12**(1): 117. |
|  |  | OSPRE (referenced in - Linzer, Baier Manwell et al. 2005) |
|  |  | Kralewski’s Instrument (referenced in - Linzer, Baier Manwell et al. 2005) |
| Safety processes | Trigger Tools | Trigger Tool for Adverse Drug Reactions  Cantor, M. N., et al. (2007). "Using trigger phrases to detect adverse drug reactions in ambulatory care notes." Quality & safety in health care **16**(2): 132-134. |
|  |  | *IHI Outpatient Adverse Event Trigger Tool^23^* |
|  |  | *1000 Lives Trigger Tool for Primary Care (Wales)*  [***www.1000livesplus.wales.nhs.uk***](http://www.1000livesplus.wales.nhs.uk) |
|  |  | *The NHS III Primary Care Trigger Tool*^36^ |
|  |  | NHS Education for Scotland Primary Care Trigger Tool  De Wet, C. and P. Bowie (2011). "Screening electronic patient records to detect preventable harm: a trigger tool for primary care." Qual Prim Care **19**(2): 115-125. |
|  |  | Development of trigger tools for surveillance of adverse events in ambulatory surgery^25^ |
|  |  | Informatics tools for the development of action-oriented triggers for outpatient adverse drug events  Mull, H. J. and J. R. Nebeker (2008). "Informatics tools for the development of action-oriented triggers for outpatient adverse drug events." AMIA Annu Symp Proc: 505-509. |
|  |  | Trigger tool for identifying adverse drug events among older adults in primary care^20^ |
|  | IT | PDA (Personal Digital Assistant) toolkits such as ‘Lexi Drugs’  Galt, K. A., et al. (2005). "Personal digital assistant-based drug information sources: potential to improve medication safety." Journal of the Medical Library Association **93**(2): 229-236. |
|  |  | CDSS (Clinical decision support systems) (ATHENA)  Michel, M., et al. (2008). "Improving Patient Safety Using ATHENA-Decision Support System Technology: The Opioid Therapy for Chronic Pain Experience Technology and Medication Safety)." |
|  |  | CDSS (Clinical decision support system )  Judge, J., et al. (2006). "Prescribers' responses to alerts during medication ordering in the long term care setting." J Am Med Inform Assoc **13**(4): 385-390. |
|  |  | CPOE (Computerised Physician Order Entry) – Retrospective Medication Profiling  Glassman, P. A., et al. (2007). "The utility of adding retrospective medication profiling to computerized provider order entry in an ambulatory care population." Journal of the American Medical Informatics Association **14**(4): 424-431. |
|  |  | NEPSI (National e-Prescribing Patient Safety Initiative)  Fischer, M. A. (2007). "The National e-Prescribing Patient Safety Initiative: removing one hurdle, confronting others." Drug Safety **30**(6): 461-464. |
|  |  | SEMI – P^34^ |
|  |  | IT-enabled systems engineering approach to monitoring and reducing ADEs  Singh, R., et al. (2012). "IT-enabled systems engineering approach to monitoring and reducing ADEs." Am J Manag Care **18**(3): 169-175. |
|  |  | Computerized Drug Renal Alert Pharmacy (DRAP) program  Bhardwaja, B., et al. (2011). "Improving prescribing safety in patients with renal insufficiency in the ambulatory setting: the Drug Renal Alert Pharmacy (DRAP) program." Pharmacotherapy **31**(4): 346-356. |
|  |  | e-Pharmacovigilance  Haas, J. S., et al. (2010). "Participation in an ambulatory e-pharmacovigilance system." Pharmacoepidemiology and Drug Safety **19**(9): 961-969. |
|  |  | IMPOVE program to detect patients at risk of drug problems  Isaksen, S. F., et al. (1999). "Estimating risk factors for patients with potential drug-related problems using electronic pharmacy data. IMPROVE investigators." Ann Pharmacother **33**(4): 406-412. |
|  | Patient Involvement | APHID (Automated Patient History Intake Device ) medication kiosk  Lesselroth, B., et al. (2009). "National patient safety goals. Using consumer-based kiosk technology to improve and standardize medication reconciliation in a specialty care setting." Joint Commission Journal on Quality & Patient Safety **35**(5): 264-270. |
|  |  | Patient knowledge of Prescriptions Instrument  Frohlich, S. E., et al. (2010). "Instrument to evaluate the level of knowledge about prescription in primary care." Rev Saude Publica **44**(6): 1046-1054. |
|  |  | GERM (Buetow 2010) (Grow relationships; Enable patients and professionals to recognise and manage patient error; be Responsive to their shared capacity for change; and Motivate them to act together for patient safety)  Buetow, S., et al. (2010). "Approaches to reducing the most important patient errors in primary health-care: patient and professional perspectives." Health & social care in the community **18**(3): 296-303. |
|  |  | *Speak Up (JCAHO^33^* |
|  |  | ACEPP (Communication about Evidence and Patient Preferences)  Shepherd, H. L., et al. (2011). "Three questions that patients can ask to improve the quality of information physicians give about treatment options: A cross-over trial." Patient Education and Counseling **84 (3)**: 379-385. |
|  |  | Safe to Ask  Byrd, J. and L. Thompson (2008). ""It's safe to ask": promoting patient safety through health literacy." Healthc Q **11**(3 Spec No.): 91-94 |
|  |  | SEAPS (Seniors Empowerment and Advocacy in Patient Safety )^32^ |
|  |  | Medication Risk Questionnaire  Langford, B. J., et al. (2006). "Implementation of a self-administered questionnaire to identify patients at risk for medication-related problems in a family health center." Pharmacotherapy **26**(2): 260-268. |
|  |  | Self-administered medication-risk questionnaire  Barenholtz Levy, H. (2003). "Self-administered medication-risk questionnaire in an elderly population." Ann Pharmacother **37**(7-8): 982-987 |
|  |  | Appropriateness of Prescribing  Britten, N., et al. (2003). "Developing a measure for the appropriateness of prescribing in general practice." Quality & safety in health care **12**(4): 246-250. |
|  | Diagnosis | Gut feelings consensus  Stolper, E., et al. (2009). "Consensus on gut feelings in general practice." BMC Fam Pract **10**: 66. |
|  |  | *DxPlain^35^* |
|  |  | ISABEL  Ramnarayan, P., et al. (2004). "ISABEL: a novel approach to the reduction of medical error." Clinical Risk(Jan). |
|  |  | Electronic screening for diagnostic error (*Singh et al -* [*see*](#_ENREF_36) *appendix 2)* |
|  | Referral | Electronic Referral Tool  Gandhi, T. K., et al. (2008). "Improving Referral Communication Using a Referral Tool Within an Electronic Medical Record Performance and Tools." |
|  |  | *AHRQ criteria on referrals http://www.ahrq.gov/* |
|  |  | Out Of Hours indicators for referring and prescribing  Giesen, P., et al. (2007). "Out-of-hours primary care: development of indicators for prescribing and referring." International Journal for Quality in Health Care **19**(5): 289-295. |
|  | Interface | LIMM (Landskrona Integrated Medicines Management ) – a hospital tool with learning for family practitioners  Bergkvist, A., et al. (2009). "Improved quality in the hospital discharge summary reduces medication errors--LIMM: Landskrona Integrated Medicines Management." European Journal of Clinical Pharmacology **65**(10): 1037-1046. |
|  |  | *AHRQ (*Agency for Healthcare Research and Quality *) criteria http://www.ahrq.gov/* |
|  |  | *JCAHO (*Joint Commission on Accreditation of Healthcare Organizations *) targets http://www.jointcommission.org/standards_information/standards.aspx* |
|  |  | The Partners Post-Discharge Medication Reconciliation Tool^31^ |
|  |  | *The Medications Discrepancy Tool (*[*http://www.caretransitions.org/mdt_main.asp*](http://www.caretransitions.org/mdt_main.asp)*)* |
|  |  | *Interface and Medications Reconciliation bundles*  *<http://www.healthcareimprovementscotland.org/our_work/patient_safety/spsp_primary_care_resources/medicine_reconciliation.aspx>* |
|  |  | Physician Prepared – GP score  Graumlich, J. F., et al. (2008). "Discharge Planning Scale: Community Physicians' Perspective." Journal of Hospital Medicine **3**(6): 455-464 |
|  | Prescri-bing indicators | STOPP/START indicators (and papers which draw on them)  O'Mahony, D., et al. (2010). "STOPP & START criteria: A new approach to detecting potentially inappropriate prescribing in old age." European Geriatric Medicine **1 (1)**: 45-51. |
|  |  | Beers Indicators (and papers which draw on them)^20^ |
|  |  | Royal College of General Practitioners indicator set  Avery, A. J., et al. (2011). "Development of prescribing-safety indicators for GPs using the RAND Appropriateness Method." British Journal of General Practice **61**(589): e526-536. |
|  |  | Guthrie Indicators  Guthrie, B., et al. (2011). "High risk prescribing in primary care patients particularly vulnerable to adverse drug events: cross sectional population database analysis in Scottish general practice." BMJ **342**: d3514. |
|  |  | PINCER trial indicators  Avery, A. J., et al. (2012). "A pharmacist-led information technology intervention for medication errors (PINCER): a multicentre, cluster randomised, controlled trial and cost-effectiveness analysis." Lancet **379**(9823): 1310-1319. |
|  |  | Drug Related Morbidity Indicators  Morris, C. J. and J. A. Cantrill (2003). "Preventing drug-related morbidity -- the development of quality indicators." Journal of Clinical Pharmacy & Therapeutics **28**(4): 295-305 |
|  |  | Wessell Primary Care Indicators  Wessell, A. M., et al. (2010). "Medication prescribing and monitoring errors in primary care: a report from the Practice Partner Research Network." Quality & safety in health care **19**(5): e21-e21. |
|  |  | NORGEP (Norwegian General Practice) indicators  Rognstad, S., et al. (2009). "The Norwegian General Practice (NORGEP) criteria for assessing potentially inappropriate prescriptions to elderly patients." Scandinavian journal of primary health care **27**(3): 153-159. |
|  |  | General Practice indicator set  Williams, D., et al. (2005). "The application of prescribing indicators to a primary care prescription database in Ireland." European Journal of Clinical Pharmacology **61 (2)**: 127-133. |
|  |  | Zhan Indicators  Zhan, C., et al. (2005). "Suboptimal prescribing in elderly outpatients: potentially harmful drug-drug and drug-disease combinations." Journal of the American Geriatrics Society **53**(2): 262-267. |
|  |  | Ten nursing home indicators  Bergman, A., et al. (2007). "Evaluation of the quality of drug therapy among elderly patients in nursing homes." Scandinavian journal of primary health care **25**(1): 9-14. |
|  |  | Lab Safety Monitoring  Raebel, M. A., et al. (2006). "Randomized trial to improve laboratory safety monitoring of ongoing drug therapy in ambulatory patients." Pharmacotherapy **26**(5): 619-626. |
|  |  | Drug/lab pairs  Yu, S., et al. (2011). "Selection of drug-laboratory result pairs for an inpatient asynchronous alert program: Results of a Delphi survey." American Journal of Health-System Pharmacy **68 (5)**: 407-414. |
|  |  | NSAID (non-sterolidal anti-inflammatory drugs) indicators  Fernandez Urrusuno, R., et al. (2008). "Development of NSAIDs prescription indicators based on health outcomes." European Journal of Clinical Pharmacology **64**(1): 61-67. |
|  |  | HIC (Health Insurance Commission) indicators  Robertson, H. A. and N. J. MacKinnon (2002). "Development of a list of consensus-approved clinical indicators of preventable drug-related morbidity in older adults." Clin Ther **24**(10): 1595-1613. |
|  |  | Canadian Indicators  McLeod, P. J., et al. (1997). "Defining inappropriate practices in prescribing for elderly people: a national consensus panel." CMAJ **156**(3): 385-391. |
|  |  | IPET (improved prescribing in the elderly tool)  Naugler, C. T., et al. (2000). "Development and validation of an improving prescribing in the elderly tool." Can J Clin Pharmacol **7**(2): 103-107. |
|  |  | Australian Elders Indicators  Basger, B. J., et al. (2008). "Inappropriate medication use and prescribing indicators in elderly Australians: development of a prescribing indicators tool." Drugs Aging **25**(9): 777-793. |
|  |  | French consensus indicators  Laroche, M. L., et al. (2007). "Potentially inappropriate medications in the elderly: a French consensus panel list." Eur J Clin Pharmacol **63**(8): 725-731. |
|  |  | Which prescribing indicators do GPs prefer?  Rasmussen, H. M., et al. (2005). "General practitioners prefer prescribing indicators based on detailed information on individual patients: a Delphi study." Eur J Clin Pharmacol **61**(3): 237-241. |
|  |  | Domains of drug appropriateness  Tully, M. P. and J. A. Cantrill (2002). "Exploring the domains of appropriateness of drug therapy, using the Nominal Group Technique." Pharmacy World & Science **24**(4): 128-131. |
|  | Other prescribing tools | Prescription Optimization Method – an educational tool  Drenth-van Maanen, A. C., et al. (2009). "Prescribing optimization method for improving prescribing in elderly patients receiving polypharmacy: results of application to case histories by general practitioners." Drugs Aging **26**(8): 687-701. |
|  |  | MIQUEST (Morbidity Information Query and Export Syntax) software  Hammersley, V. S., et al. (2006). "Applying preventable drug-related morbidity indicators to the electronic patient record in UK primary care: methodological development." J Clin Pharm Ther **31**(3): 223-229 |
|  |  | GRAM tool  Lapane, K. L., et al. (2011). "Effect of a pharmacist-led multicomponent intervention focusing on the medication monitoring phase to prevent potential adverse drug events in nursing homes." J Am Geriatr Soc **59**(7): 1238-1245. |
|  |  | Medications Appropriateness Index  Hanlon, J. T., et al. (1992). "A method for assessing drug therapy appropriateness." J Clin Epidemiol **45**(10): 1045-1051 |
|  |  | PCNE - Pharmaceutical Care Network Europe classification of drug errors  Eichenberger, P. M., et al. (2010). "Classification of drug-related problems with new prescriptions using a modified PCNE classification system." Pharm World Sci **32**(3): 362-372. |
|  |  | *1000 Lives Improving Medications Management (Wales)*  [***www.1000livesplus.wales.nhs.uk***](http://www.1000livesplus.wales.nhs.uk) |
|  |  | Wise List  Gustafsson, L. L., et al. (2011). "The 'Wise List' - A Comprehensive Concept to Select, Communicate and Achieve Adherence to Recommendations of Essential Drugs in Ambulatory Care in Stockholm." Basic & Clinical Pharmacology & Toxicology **108**(4): 224-233. |
|  |  | Black box warnings  Yu, D. T., et al. (2011). "Impact of implementing alerts about medication black-box warnings in electronic health records." Pharmacoepidemiology & Drug Safety **20**(2): 192-202. |
| Safety Outcomes | Reporting systems | RAID (Recognising the acutely ill and deteriorating patient)  Evans, A., et al. (2007). "Incident reporting improves safety: the use of the RAID process for improving incident reporting and learning within primary care." Quality in Primary Care **15**(2): 107-112. |
|  |  | *National Reporting Learning System (NPSA)*  *http://www.nrls.npsa.nhs.uk/report/* |
|  |  | Jeder Fehler Zaehlt – every error counts  Hoffmann, B., et al. (2008). ""Every error counts": a web-based incident reporting and learning system for general practice." Quality & safety in health care **17**(4): 307-312 |
|  |  | Spiegel – mirror  Zwart, D. L., et al. (2011). "Feasibility of centre-based incident reporting in primary healthcare: the SPIEGEL study." BMJ Qual Saf **20**(2): 121-127. |
|  |  | ASIPS (Applied Strategies for Improving Patient Safety)  Fernald, D. H., et al. (2004). "Event reporting to a primary care patient safety reporting system: a report from the ASIPS collaborative." Ann Fam Med **2**(4): 327-332. |
|  |  | Error reporting system and taxonomy  Rubin, G., et al. (2003). "Errors in general practice: development of an error classification and pilot study of a method for detecting errors." Quality & safety in health care **12**(6): 443-447. |
|  |  | *Yellow Card System (MHRA)*  *http://yellowcard.mhra.gov.uk/* |
|  |  | *Device Safety reporting (MHRA)*  *http://www.mhra.gov.uk/Safetyinformation/Reportingsafetyproblems/Devices/* |
|  |  | MEADERS ^27^ |
|  |  | VAERS (Vaccine Adverse Event Reporting System)*^28^* |
|  | Significant Event Analyses | Significant Event appraisal  McKay, J., et al. (2007). "Development and testing of an assessment instrument for the formative peer review of significant event analyses." Quality & safety in health care **16**(2): 150-153. |
|  |  | *Quality and Outcomes Framework system for Significant Event Analyses – withdrawn since this SR undertaken* |
|  |  | System for improving SEA (*Significant Event Analyses* ^)29^ |
|  |  | *SEA (Significant Event Analyses )– a practical guide (Medical Defence Union - UK)*  *[www.mdu.org.uk](http://www.mdu.org.uk)* |
|  | User Evaluation | Disclosing Medical Error to patients  Wu, A. W., et al. (2009). "Disclosing medical errors to patients: it's not what you say, it's what they hear." J Gen Intern Med **24**(9): 1012-1017. |
|  |  | Measuring Patient Safety by Patient Experiences  Solberg, L. I., et al. (2008). "Can patient safety be measured by surveys of patient experiences?" Jt Comm J Qual Patient Saf **34**(5): 266-274 |
|  |  | Patient Survey of Medical Error  Blendon, R. J., et al. (2002). "Views of practicing physicians and the public on medical errors." N Engl J Med **347**(24): 1933-1940. |

**Web Appendix 4. Taxonomy**

**The operational framework for primary care patient safety**

**Safety structure & systems Processes of safety Outcomes of ‘safety’**

^1^Availability ^1^Availability  ^12^ User evaluation

^2^Background systems inc ^10^ clinical care ^13^ Mortality

^3^ Informatics & interface ^11^ interpersonal care ^14^ Adverse events (harm)

^4^Management ^15^ Errors

^5^ Premises ^16^ Incident reports

^6^ Workforce/team ^17^ Significant events ^7^ System interface  ^18^ Improvement

^8^ Learning organisation

^9^ Patient / carer role

Safety structure and systems

**^1^Availability** includes: organizational access; continuity (informational), service availability/timeliness; systems of access, triage; emergency access; information for patients on access;

^2^ **background systems**: flow of data/information/ data handling; clinical handover (referral, discharge summaries structures and responses), preventing harm risk registers [including children at risk; palliative care]; trigger tools [i.e. NHS Institute 24 item primary care trigger tool]; information on named carer; reporting [including incidents; complaints]; response to abnormal test results and follow up to results & investigations;

^3^ **Electronic health record/informatics** includes: prompts/triggers; documentation; interface; prescribing/dispensing; (CPOE [computerized physician order entry systems];

^4^**Management** includes: governance; drug safety [including in stock and expiry]; infection control; patient information leaflets; monitoring of staff roles/ qualifications; hazard protocols [any threat to safety, e.g. unsafe practices, conduct, labels etc]

^5^ **Premises** includes: equipment, devices, car parking if on site, health and safety;

^6^ **Workforce/team** includes: skills, training, qualifications, communication, responsibilities/lines of authority, workload monitoring, occupational health including monitoring fatigue/burnout;

^7^ **System interface** includes: information exchange and partnership working; out-of-hours; pharmacies;

^8^ **Learning organization** includes: knowing and responding to the needs of the practice population/ community; safety culture/climate; adherence to protocols/guidance; SEA; attitudes to patent safety; CPD; training; evidence of individual’s up-to-date training; patient involvement;

^9^ **Patient/carer role** **& involvement**: including patient participation;

Safety processes

^10^ Clinical includes:

**Diagnoses**, including missed and delayed diagnoses

**Problem and needs management**, including new patient checks; contraindications and allergies; multimorbidity; multiple medications; frailty; palliative care;

**Investigations**, including laboratory tests and results,

**Prescribing**: including preventing harm and errors

**Treatment**: including minor surgery

**Follow-up**: including diarised activity,

**Coordination**,

**Referral**,

**Discharge**,

**Interface** including patient transfer

**Pathways**:

^11^ Inter-personal includes:

**communication**,

**monitoring**,

**inter-personal continuity**.

Safety outcomes

^12^ **User evaluation** includes: complaints, feedback from patients

^13^ **Mortality**

^14^ **Adverse events** (Harm)

^15^ **Errors**

^16^ **Incident reports**

^17^ **Significant events** (audits)

^18^ **Improvement**: evidence of improvement in patient safety structures and processes, errors/adverse events and complaints etc

**Web appendix 5: Modified data extraction sheet**

Excel column1: Report ID + Reference (direct from endnote)

**STUDY CHARACTERISTICS**

Excel Column 2: Setting

1. General practice
2. Primary-secondary interface
3. OOH
4. Pharmacies
5. General practice & pharmacy
6. General practice and OOH
7. GP and dentistry
8. Nursing homes
9. Nursing homes and general practice
10. Nursing homes and pharmacies
11. Primary care education per se
12. Health system
13. Other …………………………………….(free text)

Excel column 3: Country

1) UK

2) More than one country

One country but other than UK ………………………… (create a coding scheme as go along)

1. Germany
2. Netherlands
3. USA

Excel column 4: Type of study/study design

1. Qualitative
2. Quantitative - medical record review
3. Quantitative – GPRD, database analyses
4. Systematic review – quantitative
5. Systematic review – qualitative
6. Systematic review - mixed
7. RCT
8. Observational (inc questionnaires/surveys)
9. Longitudinal cohort
10. Mixed methods
11. Consensus techniques
12. Opinion / recommendations
13. Other……………………………………………………………….(Free Text)

Excel column 5: Existing indicator-Toolkit

Is there a clearly defined indicator / set of indicators or a clearly defined/validated Tool as an aim/outcome?

1. Yes: Toolkit (electronic data extraction)
2. Yes: Toolkit (paper based or mixed data extraction : NOT just electronic)
3. Yes: PROM/PREM
4. Yes: Questionnaire
5. Yes: specific indicator or set of indicators or RAND Appropriateness Method criteria etc but not a named toolkit
6. No: BUT an indicator or indicators could be developed from the findings/outcome
7. Other: ……………………………………………………….(Free text)
8. No 🡪 EXCLUDE

Word document 5a: [word summary of why exclude]

Word document 6b: What is/are the indicator(s):………………………………………………………………

Excel column 6: What type of patient safety is assessed (topic)?

1. Diagnostic
2. Prescribing
3. Investigations
4. Referral
5. Data accuracy / informatics: internal to practice
6. Interface: primary-secondary – referral/discharge
7. Clerical error
8. OOH
9. Telephone
10. Training
11. Safety climate
12. Patient role
13. Organisational
14. Errors (generic) – no specific focus
15. Adverse events (generic) – no specific focus
16. Outcomes: mortality
17. more than one........................................................(numbers)
18. Other …………………………………………………………………………
19. Not clear

Excel column 7: Outcomes (including details of scales)

1. Indicators (as written in the paper)
2. Indicators – WE have extracted
3. Toolkit
4. Indices: e.g. medication appropriate index
5. RAND outcomes
6. PROM/PREM (patient completed questionnaire)
7. Questionnaire (not completed by patients so staff or GPs etc)
8. Taxonomy or framework
9. Other: ………………………………………………………………….……………………………….(Free text)
10. Not clear
11. None

word document 7: summary of scale:………………………………………………………………………………………..

Excel column 8: Validation/ratification

Indicators/Tool(s) ratified for use in general practice/pharmacy/primary-secondary interface?

1. Yes in general practice by research team (either as part of the study or statement/reference that this has been done previously)
2. Yes in general practice – by researchers other than research team
3. No but yes in hospital
4. No
5. Not applicable
6. Unclear

word document 8: [summary of why: ]

**APPRASIAL**

Excel column 9: aims

*( is there a clear statement of aims?)*

1. Yes
2. No
3. Not applicable
4. Unclear

word document 9: [summary of why the aims are clear]

Excel column 10: choice of method

*(Is there a statement that explains the research design (e.g. have they discussed how they decided which methods to use?).*

1. Yes
2. No
3. Not applicable
4. Unclear

word document 10: [summary of why the method is appropriate]

Excel column 11: Sampling

*Consider: is there a statement that explains how the participants were selected; If quantitative did they do a power calculation? If qualitative did they provide a statement of sampling?*

1. Yes
2. No
3. Not applicable
4. Unclear

word document 11: [summary of why sampling is appropriate]

Excel column 12 :Data collection

*( is there a statement by the researcher(s) justifying the methods chosen?)*

1. Yes
2. No
3. Not applicable
4. Unclear

word document 12: summary of why data collection is approrpiate

Excel column 13: Data analyses appropriate?

*( is there an in-depth description of the analysis process?)*

(for example, confidence intervals in quantitative; theoretical saturation in qualitative etc)

1. Yes
2. No
3. Not applicable
4. Unclear

word document 13: summary of why data analyses are appropriate

Excel column 14: results

(is there a clear statement of results?)

1. Yes
2. No
3. Not applicable
4. Unclear

word document 14: summary of why results are credible

Free text of main result……………………………………….

*Consider: a) Rigour: has a thorough and appropriate approach been applied? B) Credibility: are the findings well presented and meaningful? C) Relevance: how useful are the findings to the PST RQ?)*

Excel column 15: Strengths/limitations

(is there a discussion of the strengths/limitations of the paper?)

1. Yes
2. No
3. Not applicable
4. Unclear

word document 15: summary of a) strengths and b) weaknesses]

Excel column 16: Possible to adapt to GP clinical computer systems (existing or possible)?

1. Yes
2. No
3. Not applicable
4. Unclear

word document 16: summary of why it is adaptable to GPCS .

**Web Appendix 6:** *Grey Literature sources*

US websites; Institute for Healthcare Improvement, Joint Commission on Accreditation of Healthcare, Agency for Healthcare Research and Quality, *DxPlain* and Society to Improve Diagnosis in Medicine.

UK websites; NHS Institute for Innovation and Improvement, National Patient Safety Agency, National Prescribing Council, Medicines and Healthcare products Regulatory Authority, Medical Protection Society, Medical Defence Union, General Medical Council, 1000 lives Wales, Scottish Patient Safety Programme.

Additional information was derived from personal contacts, unpublished work and theses.
